# Psychopathological Profiles in Transsexuals and the Challenge of Their Special Status among the Sexes

**DOI:** 10.1371/journal.pone.0078469

**Published:** 2013-10-23

**Authors:** Matthias K. Auer, Nina Höhne, María Ángeles Bazarra-Castro, Hildegard Pfister, Johannes Fuss, Günter K. Stalla, Caroline Sievers, Marcus Ising

**Affiliations:** 1 Department of Internal Medicine, Endocrinology and Clinical Chemistry, Max Planck Institute of Psychiatry, Munich, Germany; 2 Department Molecular Psychology, Max Planck Institute of Psychiatry, Munich, Germany; 3 Department of Psychiatry and Psychotherapy, Central Institute of Mental Health, Medical Faculty Mannheim/University of Heidelberg, Mannheim, Germany; McGill University, Canada

## Abstract

**Objective:**

Investigating psychopathological profiles of transsexuals raises a very basic methodological question: are control groups, which represent the biological or the phenotypic sex, most suited for an optimal evaluation of psychopathology of transsexuals?

**Method:**

Male-to-female (MtF) (n=52) and female-to-male transsexuals (FtM) (n=32), receiving cross-sex hormone treatment, were compared with age matched healthy subjects of the same genetic sex (n=178) and with the same phenotypic sex (n=178) by means of the Symptom Check List-90-Revisited instrument (SCL-90-R). We performed analyses of covariance (ANCOVA) to test for group and sex effects. Furthermore, we used a profile analysis to determine if psychopathological symptom profiles of transsexuals more closely resemble genotypic sex or phenotypic sex controls.

**Results:**

Transsexual patients reported more symptoms of psychopathological distress than did healthy control subjects in all subscales of the SCL-90-R (all p<0.001), regardless of whether they were compared with phenotype or genotype matched controls. Depressive symptoms were more pronounced in MtF than in FtM (SCL-90-R score 0.85 vs. 0.45, p = 0.001). We could demonstrate that FtM primarily reflect the psychopathological profile of biological males rather than that of biological females (r = 0.945), while MtF showed a slightly higher profile similarity with biological females than with biological males (r = 0.698 vs. r = 0.685).

**Conclusion:**

Our findings suggest that phenotypic sex matched controls are potentially more appropriate for comparison with the psychopathology of transsexual patients than are genetic sex matched controls.

## Introduction

Gender identity disorder (GID), also referred to as transsexualism, is defined as a condition in which a person with apparently normal somatic sexual differentiation is convinced that he or she is actually a member of the opposite sex. This conviction is accompanied by the unchangeable and irresistible urge to live in the other sex, both physically and socially, and by the wish to acquire the physical characteristics of the desired sex to the fullest extent possible (according to ICD-10 [1] and DSM-IV criteria [2]). Biological causes of this phenomenon have been discussed before [3-5].

Treatment protocols for patients with GID follow international consensus statements [6] but vary considerably between different countries and centers. Aside from sex reassignment surgery, cross-sex hormone treatment has an important role to play in acquiring the secondary sex characteristics of the desired sex. In male-to-female- transsexuals, estradiol is usually combined with an anti-androgen such as spironolactone or cyproterone acetat for the control of testosterone levels, while female-to-male transsexuals are treated by either testosterone alone or combined with GNRH-analogues in case of in the event of persistent bleedings [6]. 

The limited data on the long-term mortality of transsexuals reveals that MtF transsexuals suffer from an increase of 51% in mortality compared to the general male population. This can mostly be attributed to a six-fold increase in suicides which is accompanied by a higher rate of psychiatric conditions [7-9], and different risk behavior leading to HIV infections as well as drug abuse [7,10]. In contrast, according to different studies, female-to-male transsexuals do not implicitly have any higher mortality risk, and may also lack an increased incidence of psychiatric disorders [11] or suicidal intentions [7]. Nonetheless there is no definite consent yet which may be the causes or predictors of an increased risk of developing depressive or anxiety disorders in the course of the treatment period, and reliable markers identifying certain high-risk individuals in terms of psychopathology are still lacking. This brings attention to the methodological aspect of psychiatric assessment in the studies discussed above, and what individual methodological characteristics have to be kept in mind when interpreting the data. Usually the results of transsexual patients are compared to those of healthy age-matched control persons of the same biological sex, or to the opposite transgender sex (FtM vs. MtF) [11,12]. 

### Aims of the study

The prevalence and extent of psychopathology such as anxiety disorders and depression are highly sex-dependent [13]. Therefore an approach comparing transgender individuals to a group belonging to their biological sex rather than the sex of their sexual identity, is questionable. We conducted a study in hormone-treated transsexuals to assess their psychopathology by means of self-reported symptoms of mental disorders, and compared the results with matching control groups, representing either the biological or the phenotypic sex of transsexuals, with the aim of examining which control group would be the best choice.

## Materials and Methods

### Ethics Statement

All participating subjects gave written informed consent, and the study was approved by the local ethics committee of the Ludwig Maximilians University in Munich (132-08). 

### Subjects

200 patients with the diagnosis “transsexualism” (GID) according to ICD-10 criteria, and treated at the Endocrine Outpatient Clinic of the Max Planck Institute of Psychiatry in Munich between January 1996 and December 2007, were identified through the electronic database of the Institute and consecutively invited to participate in this study (MtF = 118, FtM = 82) in 2009. Additionally, the clinicians and psychotherapists participating in regional quality circles for transsexualism assisted in terms of contacting some of the patients. All transsexuals underwent hormone replacement therapy in the endocrine outpatient clinic of the Max Planck Institute of Psychiatry.

Our final sample consisted of 89 transsexual patients (MtF = 57, FtM = 32) aged 18 years or older, corresponding to a response rate of 44% (MtF) and 39% (FtM), respectively, a difference which was not significant. One MtF patient was excluded from the analysis due to ambiguous information about the biological sex. Four patients were excluded because they didn’t receive hormone replacement therapy. Finally we enrolled 84 transsexual patients (52 MtF, 32 FtM) in the study. 

As a control sample, we selected 336 subjects with a negative lifetime history of mental axis I disorders, including any gender identity disorders, from a large cohort, the so called Munich Antidepressant Response Signature (MARS) control sample [14]. For the 52 MtF transsexual patients we matched, according to age, 104 male (same genetic sex, genetic match) and 104 female controls (same phenotypic sex, phenotypic match). For the 32 FtM transsexual patients we matched 64 female (genetic match) and 64 male control individuals (phenotypic match). 

### Measures

Questionnaires including general questions on the course of the sexual transition phase, treatment, social background and sexuality had been evaluated previously [11]. The evaluation in terms of psychopathology was done by means of the Symptom Check List-90-Revised (SCL-90-R) inventory. The SCL-90-R is a self-report questionnaire for assessing a broad range of psychopathology symptoms [15] and has been used earlier in this context [11]. It is composed of 90 items with regard to symptoms for which the subject rates on a ﬁve-point Likert scale of 0 (none at all) to 4 (extremely), the rate of congruence of the symptom in the last seven days. The SCL-90-R consists of 9 primary symptom dimensions: somatization (SOM), obsessionality (O-C), interpersonal sensitivity (IS), depression (DEP), anxiety (ANX), hostility (HOS), phobic anxiety (PHOB), paranoid ideation (PAR), and psychoticism (PSY) as well as the Global Severity Index (GSI) that indicates the general level of psychological distress. The German Version of the questionnaire by Franke was applied [16].

### Statistical analysis

Analyses were processed using PASW statistical software (release 18.0.0, SPSS Inc., Chicago, USA). The level of significance was set to be p= .05. We calculated Cohen’s f as the effect size measure. According to Cohen’s guidelines (1988), we categorized effects as small (f< .10), medium (f ≥ .25), and large (f ≥ .40). Mean group differences in terms of demographic and clinical variables were analyzed using the Student’s t-test in the case of quantitative variables, and Fisher’s exact tests with regard to dichotomous variables. 

We performed analyses of covariance (ANCOVA) to compare transsexual patients with their matched healthy controls (HC). The factors “group” (TS or healthy controls) and “sex” (male, female) were tested for their effects on the SCL-90-R subscales (SOM, O-C, I-S, DEP, ANX, HOS, PHOB, PAR, PSY, GSI). Two separate ANCOVAs were performed, one to investigate differences between transsexuals and matched healthy controls with the same genetic sex (sex_gen), and a second with respect to controls with the same phenotypic sex (sex_phen). Age was included as a covariate in these analyses. 

Univariate ANCOVA was used to determine the differences between MtF and FtM transsexuals in the SCL-90-R symptom subscales. We analyzed every SCL-90-R subscale regarding differences in the factor sex (MtF, FtM). In this analysis, we added age, depression diagnosis, sex reassignment surgery, age at which the transsexual patients discovered their transsexuality and the duration of hormone therapy in years, as covariates.

To elucidate whether the symptom profile of transsexual patients resembles the profile of the control group of the same genetic or the same phenotypic sex, we performed a profile analysis of the total SCL-90-R outcome using Pearson correlations as proximity measure. Pairwise correlations were calculated between transsexuals (MtF, FtM) and both healthy control groups (phenotypic match, genotypic match), and averaged after Fisher-Z transformation to a general proximity score of psychopathological symptoms. 

## Results

### Patient characteristics

In the study, the mean age of MtF transsexual patients was 47.96 (SD 11.69) and 32.38 (SD 7.75) in FtM transsexuals (p<.001). The age at onset of the gender identity disorder was significantly higher in MtF (12.78, SD 9.79) than in FtM (8.81, SD 5.22) (p=.042). The average duration of hormone replacement therapy was 7.05 years (SD 8.01) in MtF transsexuals and 5.46 years (SD 4.50) in FtM transsexuals, which was not statistically significant. Twenty-one subjects in the FtM group had undergone complete sex reassignment surgery (65.6%), similar to the MtF group with 37 (66%). The majority of MtF (48.1%) received transdermal estrogens for hormonal therapy, while most of FtM were treated by intramuscular testosterone formulations (87.5%) (Table 1). 

**Table 1:** Demographics: transsexualism, education, profession.

	**TS MtF**		**TS FtM**			
	N=52		N=32			
	Mean	SD	mean		SD	p^a^
duration hormone therapy (years)	7.05	8.01	5.46		4.50	n.s.
age of onset GID	12.78	9.79	8.81		5.22	.042
	N	%	n		%	p^b^
depression diagnosis	7	14.5	5		15.6	n.s.
hormone-therapy						
oral estrogens	13	25.0	-		-	
transdermal estrogens	25	48.1	-		-	
intramuscular estrogens	8	15.4	-		-	
unknown route of application	6	11.5	-		-	
additional antiandrogens	17	23				
intramuscular testosterone	-	-	28		87.5	
transdermal testosterone	-	-	3		9.4	
unknown route of application	-	-	1		3.1	
sex reassignment surgery						n.s.
no surgery performed	16	30.1	8	25		
complete reassignment^c^	34	65.4	21	65.6		
incomplete reassignment	2	4.5	3	9.4		
orchidectomy only	1					
breast augmentation only	1					
mastectomy only			2			
hysterectomy only			1			
profession						
student	3	5.8	6	18.8		
working full time	29	55.8	19	59.4		n.s.
working part time	4	7.7	1	3.1		
retired	6	11.5	1	3.1		
Unemployed (seeking employment)	7	13.5	2	6.3		
Unemployed	3	5.8	2	6.3		
education status						
no graduation	2	3.8	0	0.0		
secondary school	17	32.7	6	18.8		n.s.
intermediate school	9	17.3	13	40.6		
technical school	8	15.4	2	6.3		
higher education	15	28.8	10	31.3		
committed relationship	18	34.6	14	43.8		n.s.

Note: TS MfF = male-to-female transsexuals; TS FtM = female-to-male transsexuals; GID = Gender Identity Disorder; SD = standard deviation; n.s. = not significant on a significance level of p<.05; SD = standard deviation.

^a^ p-value are from Students t-test

^b^ p-value are from Chi-square test or Fisher’s exact test (depression diagnosis variable)

^c^ in FtM: hysterectomy, ovariectomy, mastectomy, phalloplasty; in MtF bilateral orchidectomie, penectomy, breast augmentation if necessary, vaginoplasty, vulvoplasty

There was no significant difference in terms of the highest education level or the current employment situation. Fourteen (43.8%) of the FtM transsexuals lived in a committed relationship in comparison to eighteen in the MtF group (34.6%), though the difference was not significant. 

In the group of MtF transsexuals, seven patients reported a diagnosis of depression in their history (13.5%) compared to five patients (15.6%) in FtM transsexuals (p=.761) (Table 1,2).

**Table 2:** Demographics: SCL-90-R mean scores in TS and HC.

	**TS MtF**		**TS FtM**		**HC men gen**		**HC women**		**HC men**		**HC women**	
							**gen**		**phen**		**phen**	
	n=52		n=32		n=104		n=64		n=64		n=104	
	mean	SD	mean	SD	mean	SD	mean	SD	mean	SD	mean	SD
age	47.96	11.69	32.38	7.75	48.27	10.91	32.77	7.92	48.16	11.40	33.44	7.20
SCL-90-R												
SOM	.51	.47	.56	.50	.31	.34	.34	.33	.22	.18	.33	.30
O-C	.64	.64	.58	.56	.31	.39	.32	.31	.22	.26	.29	.31
IS	.73	.76	.59	.77	.28	.43	.24	.23	.18	.23	.27	.29
DEP	.85	.76	.45	.55	.23	.33	.31	.40	.17	.23	.26	.28
ANX	.40	.48	.36	.51	.15	.31	.19	.27	.10	.18	.18	.22
HOS	.49	.52	.46	.66	.20	.39	.23	.29	.13	.18	.21	.27
PHOB	.40	.68	.19	.42	.08	.23	.04	.09	.03	.07	.07	.13
PAR	.72	.83	.49	.66	.28	.43	.22	.27	.15	.23	.27	.35
PSY	.30	.36	.25	.35	.12	.26	.08	.13	.06	.11	.12	.18
GSI	.58	.51	.45	.48	.23	.28	.24	.22	.15	.14	.24	.20

Note: TS = transsexuals; HC = healthy controls; TS MtF = male-to-female transsexuals; TS FtM = female-to-male transsexuals; HC men gen = matched healthy male controls of the same genetic gender; HC women gen = matched healthy female controls of the same genetic gender, HC men gen = matched healthy male controls of the same phenotypic gender; HC women gen = matched healthy female controls of the same phenotypic gender, SD = standard deviation. SCL-90-*R* symptom dimensions: SOM = somatization, O-C = obsessionality, IS = interpersonal sensitivity, DEP = depression, ANX = anxiety, HOS = hostility, PHOB = phobic anxiety, PAR = paranoid ideation, PSY = psychoticism, GSI= Global Severity Index.

### Univariate analysis of SCL-90-R subscales in transsexuals (MtF vs. FtM)

By comparing MtF and FtM transsexuals in the mean with regard to SCL-90-R subscales, we found a different distribution of psychopathology (see Table 2). Significant sex differences occurred in the subscales obsessionality (p=.015), depression (p=.001), phobic anxiety (p=.007), paranoid ideation (p=.022) and also in the general psychopathological scale GSI (p=.028). The strongest effect was seen in the subscale depression (f=.395), which could be categorized as a medium effect. MtF transsexuals reported significantly more symptoms of depression than did FtM transsexual patients, and also had significantly higher mean scores in the subscales obsessionality, phobic anxiety, paranoid ideation, and in the overall psychopathology index GSI. 

### Univariate analysis of SCL-90-R subscales in transsexuals compared to healthy controls (TS vs. HC)

**Figure 1:**
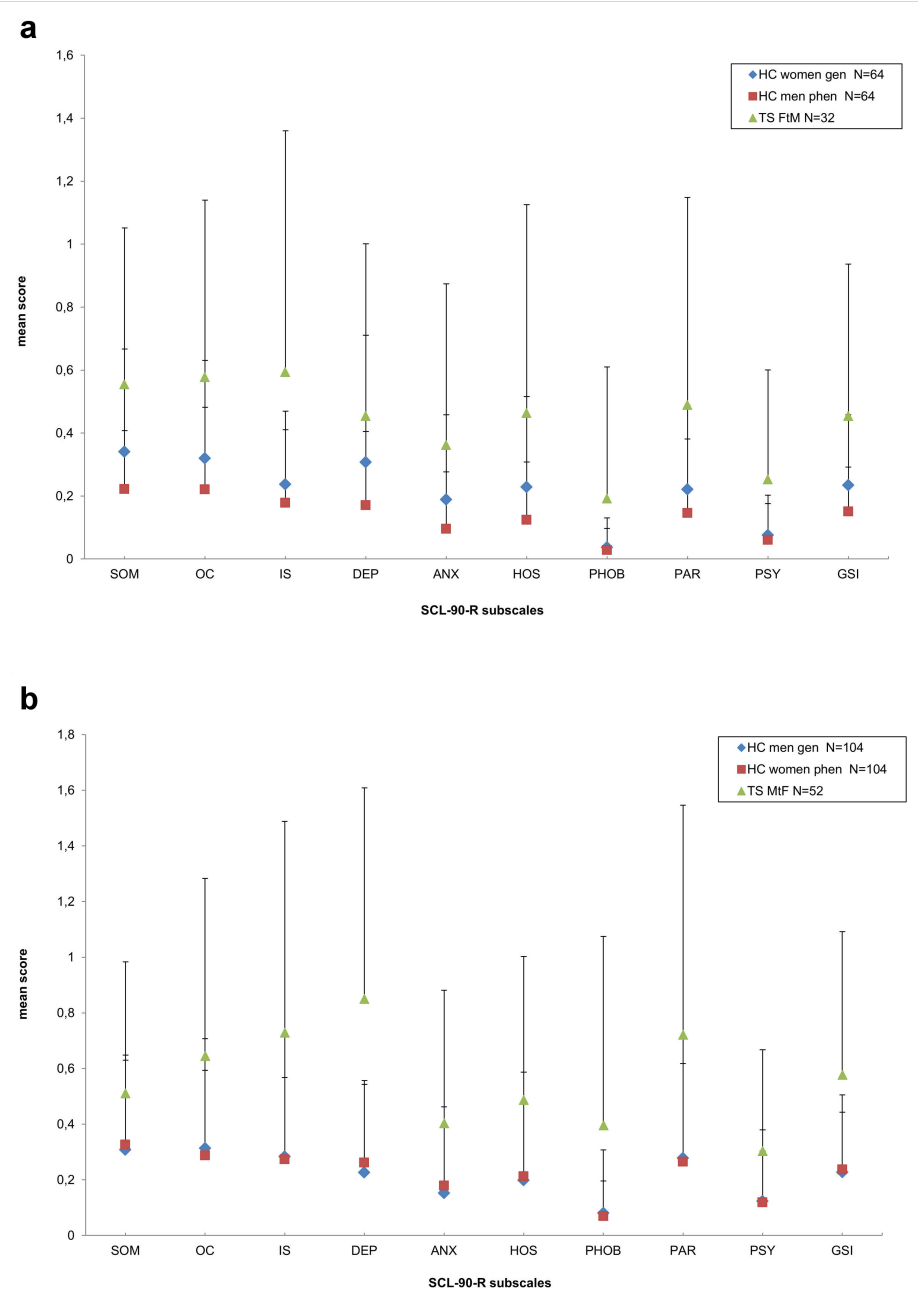
Profiles of SCL-90-R subscales in FtM and MtF transsexuals and genoptype/phenotype matched healthy controls (HC). FtM present with high scores in most SCL90 subscales. Proximity analysis revealed that profiles of FtM best resembled those of the phenotype matched male control group, but also high resemblance with the genotype matched female control group was seen (**a**). MtF present with high scores in different SCL90 subscales, most prominent in the subscale depression. MtF transsexuals had considerable, but smaller profile proximity with both control groups (**b**). Note: TS = transsexuals; HC = healthy controls; TS MtF = male-to-female transsexuals; TS FtM = female-to-male transsexuals; HC men gen = matched healthy male controls of the same genetic gender; HC women gen = matched healthy female controls of the same genetic gender; HC men gen = matched healthy male controls of the same phenotypic gender; HC women gen = matched healthy female controls of the same phenotypic gender;, SCL-90-R symptom dimensions: SOM = somatization, O-C = obsessionality, IS = interpersonal sensitivity, DEP = depression, ANX = anxiety, HOS = hostility, PHOB = phobic anxiety, PAR = paranoid ideation, PSY = psychoticism, GSI= Global Severity Index. SD = standard deviation.

The mean scores of the SCL-90-R subscales are shown in Table 2, with transsexual patients divided into MtF and FtM, and the control groups divided into genotype (sex_gen) and phenotype (sex_phen) matched healthy controls. The SCL-90-R profiles of the MtF and FtM transsexuals compared to their respective control groups of the same genetic or phenotypic sex, are presented in Figures 1a and 1b.

First, we analyzed the effects of group (transsexual vs. control) and sex (genetic men vs. genetic women) in transsexual patients, and matched healthy controls with the same genotypic sex (sex_gen). We also tested for the interaction effects in terms of group and sex. Transsexual patients had significantly higher mean scores in all SCL-90-R subscales compared to healthy controls, which was significant at p<.001 for all psychopathology subscales. Regarding sex differences in the analysis with genetic matches, we found significant main effects of sex in the following SCL-90-R-subscales: depression (p=.013), phobic anxiety (p=.001) and paranoid ideation (p=.011). The largest sex effect (f=.242) was found for the subscale phobic anxiety. There was an interaction effect in terms of group x sex_gen (p=.001) for the scale depression, with MtF patients reporting more severe depressive symptoms than FtM, as well as male and female genotype matched controls (see Table 3). 

**Table 3:** ANCOVA p-values and effect sizes for different SCL90-scores.

	**Matched for genetic gender: sex_gen**							**Matched for phenotype gender: sex_phen**					
	main effect		main effect		interaction			main effect		main effect		interaction	
	group		gender		group x			group		gender		group x	
	(TS vs. HC)		(male vs.		gender			(TS vs. HC)		(male vs.		gender	
			female)							female)			
	p	f	p	f	p	f		p	f	p	f	p	f
SOM	<.001	.248	.346	-	.913	-		<.001	.392	.845	-	.125	-
O-C	<.001	.295	.183	-	.562	-		<.001	.451	.133	-	.984	-
IS	<.001	.348	.055	-	.541	-		<.001	.477	.027	.153	.751	-
DEP	<.001	.360	.013	.172	.001	.225		<.001	.287	.000	.527	.013	
ANX	<.001	.264	.436	-	.448	-		<.001	.394	.108	-	.659	-
HOS	<.001	.274	.196	-	.651	-		<.001	.424	.035	.144	.569	-
PHOB	<.001	.291	.001	.242	.115	-		<.001	.363	.001	.227	.085	-
PAR	<.001	.305	.011	.176	.238	-		<.001	.417	.006	.190	.409	-
PSY	<.001	.303	.225	-	.965	-		<.001	.410	.173	-	.912	-
GSI	<.001	.366	.094	-	.189	-		<.001	.540	.010	.180	.673	-
SOM	<.001	.248	.346	-	.913	-		<.001	.392	.845	-	.125	-
O-C	<.001	.295	.183	-	.562	-		<.001	.451	.133	-	.984	-

Note: sex_gen = age matched healthy controls of the same genetic gender; sex_phen = age matched healthy controls of the same phenotypic gender; TS = transsexuals; HC = healthy controls. SCL-90-R symptom dimensions: SOM = somatization, O-C = obsessionality, IS = interpersonal sensitivity, DEP = depression, ANX = anxiety, HOS = hostility, PHOB = phobic anxiety, PAR = paranoid ideation, PSY = psychoticism, GSI= Global Severity Index. p = p-value, f = Cohen’s f effect size, reported if p<.05.

Next, we repeated the same analysis after exchanging the control group with healthy controls, which were matched for the phenotypic sex of the transsexual patients (sex_phen). Again, we found significant group differences in all SCL-90-R subscales (p<.001), with transsexual patients reporting significantly more psychopathology symptoms than the phenotype matched healthy control group. Significant main effects of sex appeared for the following SCL-90-R subscales: IS (p=.027), depression (p<.001), hostility (p=.035), phobic anxiety (p=.001), paranoid ideation (p=.006), and for the general psychological distress index GSI (p=.010). The strongest sex effect (f=.527) was found for the subscale depression, which could be categorized as a large effect. There was a significant interaction effect with regard to group x sex_phen (p=.013) in this subscale. Again, MtF patients reported the most severe depressive symptoms, higher than those observed in FtM, as well as in female and male subjects of the phenotype-matched controls (see Table 3, Figures 1a, b). There was no significant difference regarding any subscale of the SCL-90-R between MtF taking additional antiandrogens and those who did not (data not shown).

### Profile analysis

Next, we conducted a profile analysis to determine whether psychopathological symptom profiles resemble the profile of controls with the same genotypic or phenotypic sex. Separate profile correlations between TS (MtF, FtM) and healthy controls of the same genotypic or phenotypic sex were conducted across all SCL-90-R subscales. FtM showed the closest proximity with the phenotype-matched male control group (r=.945), but also showed a high resemblance with the genotype-matched female control group (r=.895). Also MtF transsexuals had considerable, but smaller profile proximity with both control groups, with an average correlation of r=.685 with the genetically-matched control group of males and of r=.698 with the phenotypically-matched female control group.

## Discussion

In this study, we could show that transsexual patients present with significantly higher mean scores in all psychopathology subscales of the SCL-90-R, regardless of whether they are compared with phenotype- or genotype-matched controls. The effects ranged between a medium and a large effect size. This suggests an increased burden of psychopathological distress in transsexual patients. In particular, depressive symptoms were more pronounced in MtF than in FtM, which is in accordance with the literature. Furthermore, we could demonstrate that FtM primarily reflect the psychopathological profile of biological males rather than that of biological females, while MtF showed slightly higher profile similarities with biological females rather than with biological males.

By performing separate analyses to find the proper control group for transsexual patients, we could show that group effects were present in both comparisons, but sex effects differed, depending on the genetic or phenotypic sex of the controls. Both comparisons revealed sex effects for the SCL-90-R subscales depression, phobic anxiety and paranoid ideation, with larger effect sizes observed in the second analysis, when transsexual patients were matched with controls of the same phenotypic sex. In addition to these effects, women scored higher in the subscale obsessionality, and the general psychopathological scale GSI in the second analysis. Both analyses also revealed a group by sex interaction for the depression subscale, with MtF transsexuals reporting most depressive symptoms. This interaction effect was less pronounced in the second analysis as a result of the larger difference between FtM and phenotypic sex-matched controls regarding depressive symptoms.

Though there are several studies on psychopathology in the transgender population [17-19], so far little attention has been paid to the sex-specific rates of psychiatric disturbances among biological men and women [20]. However, when aiming to investigate psychopathological differences in the transgender population, findings depend on the selection of the right control group. Due to their special status among the sexes, transsexuals can theoretically either be compared to a control group matched for the same genetic or for the same phenotypic sex.

Therefore, to evaluate the similarity of symptom profiles between patients and controls, we performed a profile analysis across all SCL-90-R subscales. The best profile similarity with FtM was seen in the male control group (matched for the phenotypic sex, r=.945). However, similarity with the female control group (matched for the genetic sex) could also be shown, albeit to a lower degree (r=.895). Regarding the MtF group, similarities with the phenotypically (r=.698) as well as with the genetically matched control group (r=.685) were both less pronounced. This might be explained by the more advanced age of the MtF group and the matched controls, resulting in an attenuated variability, especially, within the control group. However, we again observed slightly higher similarities between MtF and phenotypically-matched controls. While the marginal difference in the similarity scores between MtF patients and both control groups hinders a definite statement about the right control group in terms of providing the optimal psychopathological comparability, it is interesting that the similarity with the phenotypic sex-matched controls was higher for both patient samples, MtF and FtM. This is in line with the finding of more pronounced sex typical effects on the SCL-90-R subscales when transsexual patients are analyzed in combination with phenotypic sex-matched controls, suggesting that the expected sex differences become more visible in combination with this control group. 

The results in this study are in accordance with earlier findings on the high prevalence of depressive symptoms in the transgender population [21,22]. However, in comparison with the results from Haraldsen et al. (2000), the group difference between patients and controls in our study was larger [11]. This may be partially attributed to the fact that our control samples were pre-selected for the absence of any mental disorder, while other studies used control samples of the general population [11]. 

Our findings also correspond well with the appearance of higher SCL-90-R psychopathology scores in female subjects [15,16]. The most consistent sex effect appeared with regard to the SCL-90-R subscale, depression. Sex differences could be observed between transsexuals and both control groups, and the sex by group interaction pointed to the high depression score observed in MtF transsexuals, who reported distinctly more depressive symptoms than FtM transsexuals. Interpreting these effects, one has to consider that although MtF transsexuals reported high values in the depression subscale, there were no differences between MtF and FtM in terms of clinically diagnosed depression. Others, however, have reported a higher prevalence of mental health problems in FtM than in MtF [19]. 

The discrepancy between SCL-90-R psychopathology scores and the history of clinical diagnosis of depression, may be explained by the way the depression diagnosis was obtained, which was from chart analysis and self-reports. This might have led to an underestimation of the true prevalence in our sample. 

In summary, it appears that within the transsexual population, MtF have a higher burden of depressive symptoms compared to healthy controls, but also compared to FtM transsexuals. This is in agreement with current research findings [8,23]. Asscheman et al. [7] demonstrated that MtF under cross-sex hormone treatment had a 51% higher mortality risk, mainly due to an increased suicide rate, than the general population. In FtM this increase was not observed. Though the increase in suicide rates in these patients is certainly multifactorial, the most obvious reason for this observation is an increased prevalence of depression. There are a variety of factors which may explain the higher rate of psychiatric conditions in MtF in comparison to FtM. These may be related to social factors such as the acceptance of this disorder by society [24] or the feeling of unattractiveness related to an incongruent body image [25]. Especially in male-to-female transsexuals, it has to be taken into account that if cross-sex hormonal therapy is not induced prior to puberty, individuals have male facial characteristics and male voice patterns which leads to a stigmatization of the individual [26]. However, a satisfying explanation for the difference between MtF and FtM in terms of psychopathology is still pending, and so far remains speculative. It may be attributed, for example, to the fact that MtF feel more stigmatized than FtM [25], potentially as result of a low social acceptance of MtF in the general population [27]. While the initiation of cross-sex hormone treatment, apart from genital characteristics, will lead to an almost perfect male phenotype in FtM, MtF often have to struggle with stigmata such as a deep voice and persistent male facial bone characteristics, clearly identifying them as being born a biological male [28].

Since the initiation of hormone treatment has been reported to have a fundamental impact on general well-being and mood [9], we decided for reasons of sample homogeneity, and to increase the statistical power, only to include those in our analysis who were already receiving cross-sex hormone treatment at the time of the evaluation. 

Although sex steroids are widely involved in mood regulation [29] and may at least partly account for differences in gender prevalences of mood disorders [30], their exact impact on the psychopathology in transsexuals, for example by direct biological effects on brain functioning and neurotransmitter metabolism [31], remains an object of speculation. 

Low testosterone levels or hypogonadism has been repeatedly associated with mood disturbances in biological males [32]. Correspondingly, in MtF, negative effects on mental health may be exerted by complete androgen withdrawal. 

However, a recent study demonstrated that cross-sex hormone treatment has beneficial effects in terms of lower levels of anxiety and depressive symptoms in FtM as well as in MtF [9]. In further studies it would therefore be interesting to see which mechanisms have an effect on mood in MtF compared to hypogonadal men. However, such features as estrogen substitution in MtF, and an especially low testosterone level, often being lower than that usually seen in premenopausal biological females [33], may interfere with result interpretation. 

In addition, there are also fundamental gender differences regarding social interactions and partnership integrity, further influencing psychological outcome measures [34]. Moreover, socioeconomic aspects such as lower income and less qualified employment on the part of MtF seem to have a role to play in this context [19]. Indeed, in our sample, unemployment tended to be more common among MtF than among FtM. However, this different was not statistically significant. 

There have also been reports that mental health improves following sex reassignment surgery [12,35,36]. One reason might be that individuals feel less insecure and unattractive [25]. However, in our study, we found no difference between those who had already undergone sex reassignment and those who had not in terms of any subscale of psychopathology. Therefore sex reassignment surgery doesn’t seem to be a major contributor to psychosocial well-being in our transsexual sample. This is in accordance with a recent systematic review and meta-analysis which concluded that although many subjects benefit from sex reassignment, there is also evidence of higher psychiatric morbidity and suicide rates following the procedure [36]. 

To the best of our knowledge, this is the first study investigating in detail the psychopathology of transsexuals and healthy controls assessed by SCL-90-R divided into phenotype and genotype matched subjects. In summary, we could demonstrate that transsexual patients report more symptoms of psychopathological distress than healthy control subjects, regardless of whether they are compared with phenotype or genotype matched controls. Symptoms of depression were increased in MtF transsexuals, and they are also more burdened in terms of other aspects of psychopathology than FtM. The proper control group for FtM transsexuals in terms of their profile similarity with regard to psychopathological symptoms, seems to be age-matched men rather than women, supporting the view that phenotypic sex-matched controls are the more appropriate choice. While the differences in profile similarity were less pronounced between MtF and both age-matched control groups, the overall observed sex effects are additionally supportive for selecting phenotypic sex -matched controls as the appropriate comparison group for evaluating psychopathology in transsexual patients under hormone replacement therapy.

### Strength and limitations

The strength of this single-center study is its well-defined study groups. However, the choice of the control samples can either be regarded as a strength or a limitation in this context. On the one hand, all controls were initially selected as healthy control groups for a depression sample and, therefore, report low scores in SCL-90-R subscales and GSI in comparison to the general population [37]. This might explain the large group effects seen in our study. On the other hand, the homogeneity of our control group with regard to mental health also allowed us to investigate the psychopathological profiles of our transgender subjects isolated from potential confounding factors. A limitation is the higher age of the MtF transsexuals, who were on average 16 years older than the FtM group. The older age of the age-matched control groups resulted in a reduced variability in terms of psychopathology scores, thus reducing the power to identify profile similarities between MtF and genetic or phenotypic sex-matched controls. Therefore, it would be interesting to repeat the analysis of psychopathological profiles in a younger sample of MtF. 
